# Age, sex and race distribution of accelerometer-derived sleep variability in US school-aged children and adults

**DOI:** 10.1038/s41598-023-49484-5

**Published:** 2023-12-13

**Authors:** Elexis Price, Xinyue Li, Yanyan Xu, Asifhusen Mansuri, William V. McCall, Shaoyong Su, Xiaoling Wang

**Affiliations:** 1 Medical College of Georgia, Augusta University, Augusta, GA USA; 2 School of Data Science, City University of Hong Kong, Kowloon, Hong Kong China; 3 Georgia Prevention Institute, Medical College of Georgia, Augusta University, Building HS-1721, Augusta, GA 30912 USA; 4 Division of Pediatric Nephrology and Hypertension, Children’s Hospital of Georgia, Medical College of Georgia, Augusta University, Augusta, GA USA; 5 Department of Psychiatry and Health Behavior, Medical College of Georgia, Augusta University, Augusta, GA USA

**Keywords:** Epidemiology, Epidemiology, Risk factors

## Abstract

Sleep variability (e.g. intra-individual variabilities in sleep duration or sleep timing, social jetlag, and catch-up sleep) is an important factor impacting health and mortality. However, limited information is available on the distribution of these sleep parameters across the human life span. We aimed to provide distribution of sleep variability related parameters across lifespan by sex and race in a national representative sample from the U.S. population. The study included 9981 participants 6 years and older from the National Health and Nutrition Examination Survey (NHANES) 2011–2014, who had 4–7 days of valid 24-h accelerometer recording with at least one day obtained during weekend (Friday or Saturday night). Of the study participants, 43% showed ≥ 60 min sleep duration standard deviation (SD), 51% experienced ≥ 60 min catch-up sleep, 20% showed ≥ 60 min sleep midpoint SD, and 43% experienced ≥ 60 min social jetlag. American youth and young adults averaged greater sleep variability compared to other age groups. Non-Hispanic Blacks showed greater sleep variability in all parameters compared to other racial groups. There was a main effect of sex on sleep midpoint SD and social jetlag with males averaging slightly more than females. Our study provides important observations on sleep variability parameters of residents of the United States by using objectively measured sleep patterns and will provide unique insights for personalized advice on sleep hygiene.

## Introduction

Adequate sleep is vital to overall human health and the importance of sufficient sleep duration is well-known^1^. However, recent research has begun to show that sleep variability, in terms of duration and timing, is also critical to optimal health^2^. Sleep variability encompasses the daily fluctuations in sleep behavior, offering a more comprehensive understanding of sleep patterns compared to a sole focus on the average value of sleep duration. Modern society commonly experiences considerable interference in sleep due to lifestyle demands which vary from day to day^3^. Furthermore, the constraints of school/work schedules and individual chronotypes drive differences in sleep patterns on weekdays compared to weekends when individuals typically try to “catch up on sleep”^3^. The difference of sleep timing on weekdays vs weekends is known as social jetlag. The variation of sleep duration and sleep timing from day to day can also be measured by the standard deviation of sleep duration (sleep duration SD) and the standard deviation of sleep midpoint (sleep midpoint SD). Inconsistency in sleep patterns has been linked to numerous negative health outcomes including cardiovascular disease, metabolic disruptions, immune system regulation, and many other adverse health conditions^4–7^. For example, large variability in sleep duration (> 1 h of sleep duration SD) has been associated with an increased risk of cardiovascular disease in older adults and suicidal ideation in young adults^4,8^. Previous studies have even indicated that irregular sleep patterns may be more strongly associated with cardiovascular disease than inadequate sleep duration itself^9,10^. The protective effects of catch-up sleep on health have also been found to be offset in individuals with habitual increased sleep variability^11^.

The variability of sleep patterns changes with age, and a nationally representative sample is required to estimate these changes across the lifespan unbiasedly, including possible modifying demographic factors such as sex and race. A recent nationally representative cross-sectional analysis which assessed the prevalence of social jetlag among US adults using self-report questionnaire data found that almost half averaged at least 1 h of social jetlag^12^, indicating the high prevalence of sleep irregularity in US adults. While limited by variations in methodologies, a recent systematic review comprising 53 studies involving participants aged 18 and above assessed the extent to which daily intraindividual variability (IIV) in sleep timing, duration, and quality were associated with non-sleep related factors^13^. From an analysis of data extracted from 15 of these studies, it was discerned that older age was linked to reduced variability in sleep timing, while minority race was associated with increased sleep duration variability^13^. However, the evaluation of the relationship between sex and sleep/wake IIV yielded inconclusive results, likely due to constraints posed by a limited sample size^13^. Like this study, most existing research on sleep variability primarily involves adults, with limited attention given to children and adolescents. However, it is important to recognize that sleep variability may have significant implications in childhood development. A recent international systematic review explored six different sleep IIV parameters, including duration IIV and onset IIV, in children and adolescents^14^. This review identified a consistent trend such that as age increased, there was an increase in sleep duration IIV and onset/waking IIV. This pattern perhaps suggests that adolescence may be a critical period in which sleep variability exerts a notable impact.

Moreover, within this study inconsistent associations were seen between sleep variability in childhood and adolescence and various health conditions such as obesity and neurodevelopmental disorders, emphasizing the need for more standardized and consistent data measurements in future research endeavors. It is also worth noting that none of the studies included directly compared sleep IIV across different age groups which underscores the importance of further literature to investigate sleep variability across differing age groups.

All in all, national survey data on objective measurements of sleep variability is limited. More recently, increasing the usage of rest activity data provided by research-grade activity monitors (i.e. accelerometers) in population studies allows for more objective estimates of sleep parameters that can be used to measure multiple parameters of sleep variability. In the current study, using nationally representative accelerometer data from the National Health and Nutrition Examination Survey (NHANES) from 2011 to 2014, we aimed to evaluate the distribution of sleep variability across the lifespan using objectively obtained multiple sleep variability parameters in 9981 participants aged 6 years and older.

## Method

### Study population

**Figure 1:**
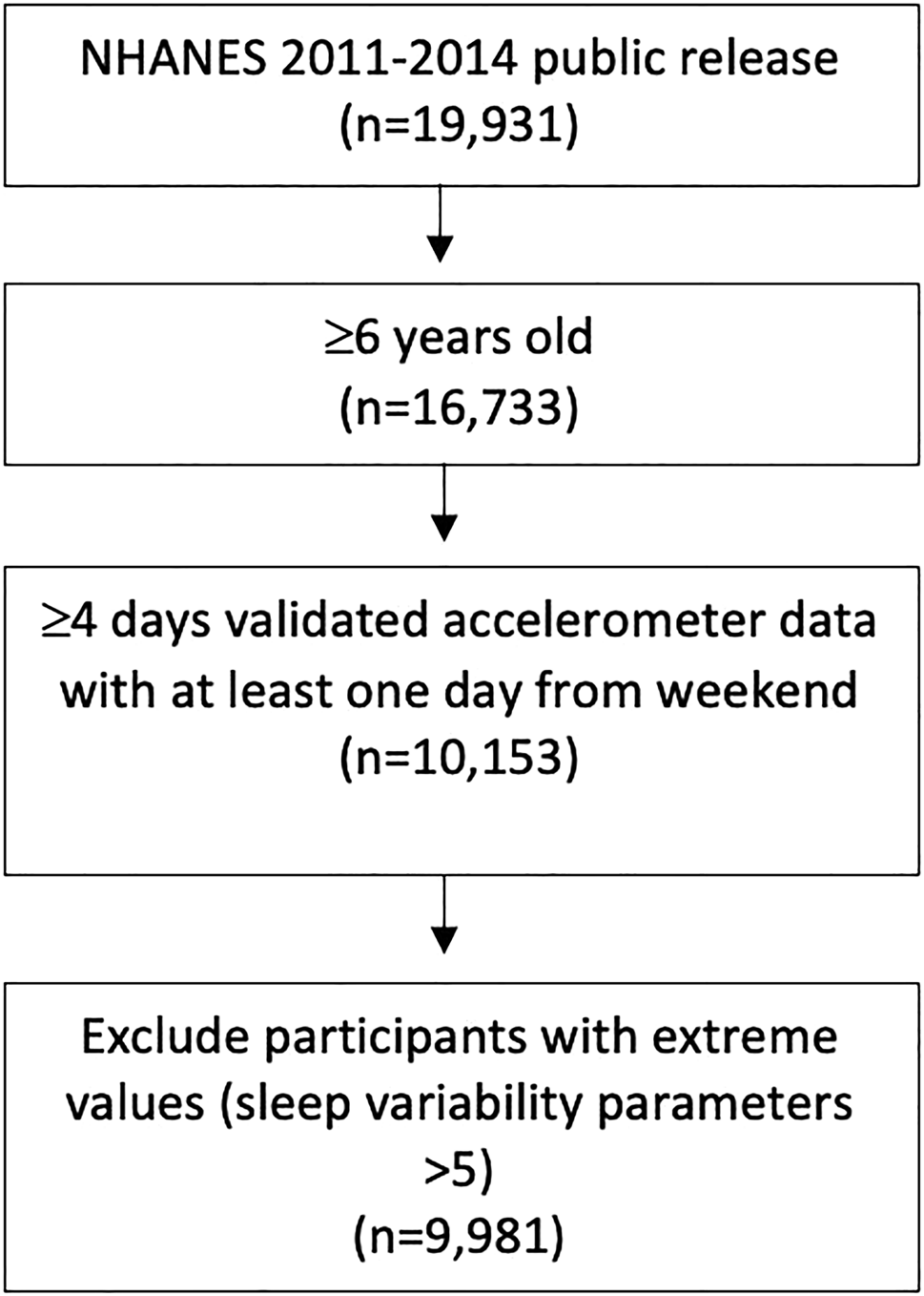
Flowchart for inclusion of study participants.

This study used data from the NHANES survey. The survey is the collection of health examination data for a weighted nationally representative sample of the U.S. population through a multistage probability sampling design^15^. The survey consists of questionnaires administered at the participants home followed by a health examination in a mobile examination center^15^. Participants receive compensation and a report of their medical findings^15^. The NHANES surveys from the 2011–2012 and 2013–2014 were used. These cycles were selected due to their availability of 24 h accelerometer data. The National Center for Health Statistics Research Ethics Review Board approved all NHANES protocols, and written informed consent was obtained from all participants or their parents or guardians. All methods were performed in accordance with the relevant guidelines and regulations. Our analytic sample (n = 9981) was selected from participants with age ≥ 6 years old (n = 16,733) that had (1) 4–7 days of valid 24-h accelerometer recording with at least one day obtained during weekend (Friday or Saturday night) (n = 10,153), and (2) all sleep variability parameters < 5 (n = 9981). NHANES does not specify participant’s age after 80 years old for participant privacy, so all participants over 80 were coded as 80 years old. Figure 1 illustrates the flow chart for participants selected for inclusion within the analysis. In comparison with the final sample (*n* = 9981), the excluded participants (*n* = 6752) were younger and less likely to be female or Non-Hispanic (NH) Whites (Supplementary Table S1). Also in comparison with the final sample, the excluded participants due to extreme sleep variability parameters (n = 172) were also likely to be NH Whites (Supplementary Table S1).

### Sleep parameters

Sleep parameters were derived from accelerometer data. Accelerometer recordings and data preprocessing were documented previously^16^ and summarized in the Supplementary document. The calculation of sleep onset time, wakeup time, and sleep duration from the accelerometer data has also been described previously. Briefly, a Hidden Markov Model (HMM)-based unsupervised sleep–wake identification algorithm was used to infer the sequence of “hidden states” of sleep or wake for each individual^17^. The block of the longest sleep period in the day (noon-noon) was identified as the sleep period time (SPT) window. The start of the SPT window was defined as the sleep onset time and the end of the SPT window was defined as the wakeup time. Wake/activity bouts were identified during the SPT window. Sleep duration was defined using the following equation: sleep duration = the SPT window duration –the summed duration of all wake bouts. The sleep midpoint was assessed by the sleep onset time and wakeup time. The variability parameters for sleep duration included the standard deviation of sleep duration (sleep duration SD) and catch-up sleep (the absolute value of the difference in sleep duration between the average of weekdays and the average of weekends). The variability parameters for timing of sleep included the standard deviation of the sleep midpoint (sleep midpoint SD) and social jetlag (the absolute value of the difference in sleep midpoint between the average of weekdays and the average of weekends).

### Age, sex, and race

The NHANES demographic file was used to obtain age, sex, and race information. Participants were divided into 10 different age groups (i.e. 6–9, 10–13, 14–17, 18–25, 26–35, 36–45, 46–55, 56–65, 66–75, and ≥ 75 years) for the description of the distributions of sleep variability parameters. Race was divided into 4 classifications: Non-Hispanic (NH) White, NH Black, Mexican American, and other race (i.e. other Hispanic, Asian and other ethnicity).

### Statistical analysis

Analyses were completed using the survey data analysis in STATA (v16) to generate representative estimates of the US population and account for complex survey design. Four-year survey weights were calculated and used in all analyses to adjust for unequal selection probability and non-response bias in accordance with NHANES analytical guidelines^18^. Population means, proportions, and standard deviation (SD) were estimated and reported. Survey weighted linear regression was used to assess the distribution of sleep variability parameters across age, sex and race. Due to the nonlinear relationship between age and sleep variability parameters, linear, quadratic, cubic and quartic trends were fitted by including age, age^2^, age^3^ and age^4^ in the regression model to understand the changes of sleep variability parameters with age. The interactions between sex and age (i.e. linear, quadratic, cubic and quartic) or between race and age were also tested to examine whether the associations of sleep variability parameters with age were modified by sex or race. Statistical significance was set at *p* < 0.05.

We performed a sensitive analyses by repeating the analyses in the participants with at least 6 days accelerometer data (86.3% of the participants) to exclude the potential bias from the inclusion of a small percentage of the participants with a relatively short period of accelerometer recording.

## Results

The complete dataset consisted of 19,931 participants with 16,733 aged ≥ 6 years. A total of 6752 participants were further excluded from analysis based on exclusion criteria (Fig. 1). Our final sample size included 9981 participants aged ≥ 6 years (mean ± SD: 41.9 ± 21.5 years), representing 178.8 million noninstitutionalized residents of the United States. The general characteristics of age, sex, race, and sleep variability parameters of participants are presented in Table 1.

**Table 1:** General characteristics of the participants (n = 9981).

Variables	Values
Age, years	41.9 ± 21.5
Female, %	53.3
Race, %	
NH White	65.3
NH Black	11.0
Mexican American	10.0
Other race	13.7
Sleep duration SD, hours	0.98 ± 0.49
Catch-up sleep, hours	1.27 ± 1.02
Midpoint of sleep SD, hours	0.72 ± 0.43
Social jetlag, hours	1.08 ± 0.92

For continuous traits, data are present as mean ± SD.

NH Non-Hispanic.

Correlations among these sleep variability parameters are listed in Supplementary Table S2. As expected, strong correlations were observed between the two parameters for day-to-day deviations in sleep duration (r = 0.52 between sleep duration SD and catch-up sleep) as well as between the two parameters for day-to-day deviations in sleep timing (r = 0.64 between sleep midpoint SD and social jetlag).

The distributions of sleep duration SD, catch-up sleep, sleep midpoint SD and social jetlag by age groups are presented in Supplementary Table S3 and Table S4. The medians ranged from 42 to 67 min for sleep duration SD, 49–82 min for catch-up sleep, 31–47 min for sleep midpoint SD and 32–86 min for social jetlag across the age groups, with the peak values shown either in the age group of 14–17 or the age group of 18–25. In the overall sample, 43% of US school-aged children and adults showed ≥ 60 min sleep duration SD, 51% experienced ≥ 60 min catch-up sleep, 20% showed ≥ 60 min sleep midpoint SD, and 43% experienced ≥ 60 min social jetlag. The prevalence of sleep irregularity defined by the cutoff of 60 min, 90 min and 120 min by these 4 sleep variability parameters by age groups are presented in Tables 2 and 3.

**Table 2:** Prevalence of sleep irregularity by cutoffs of sleep duration SD or cutoffs of catch-up sleep in different age groups (n = 9981).

Age Group	N	Sleep duration SD ≥ 1 h	Sleep duration SD ≥ 1.5 h	Sleep duration SD ≥ 2 h	Catch-up sleep ≥ 1 h	Catch-up sleep ≥ 1.5 h	Catch-up sleep ≥ 2 h
6–9	1239	16%	3%	0.3%	37%	20%	10%
10–13	1038	32%	9%	2%	49%	29%	15%
14–17	731	53%	16%	4%	59%	45%	32%
18–25	886	58%	24%	9%	60%	43%	28%
26–35	996	46%	17%	4%	55%	37%	25%
36–45	1086	43%	13%	2%	55%	37%	22%
46–55	1153	46%	16%	4%	51%	35%	25%
56–65	1240	42%	13%	3%	49%	31%	18%
66–75	932	38%	12%	3%	44%	26%	15%
≥ 76	680	40%	13%	3%	42%	27%	19%

**Table 3:** Prevalence of sleep irregularity by cutoffs of sleep midpoint SD or cutoffs of social jetlag in different age groups (n = 9981).

Age Group	N	Sleep midpoint SD ≥ 1 h	Sleep midpoint SD ≥ 1.5 h	Sleep midpoint SD ≥ 2 h	Social jetlag ≥ 1 h	Social jetlag ≥ 1.5 h	Social jetlag ≥ 2 h
6–9	1239	8%	1%	0.1%	36%	19%	10%
10–13	1038	19%	3%	0.3%	56%	36%	20%
14–17	731	28%	9%	2%	63%	47%	28%
18–25	886	33%	11%	2%	50%	35%	23%
26–35	996	26%	8%	2%	50%	34%	21%
36–45	1086	22%	6%	1%	42%	27%	17%
46–55	1153	21%	5%	2%	46%	29%	15%
56–65	1240	17%	4%	1%	36%	19%	11%
66–75	932	14%	4%	1%	26%	13%	7%
≥ 76	680	13%	3%	1%	27%	11%	4%

**Figure 2:**
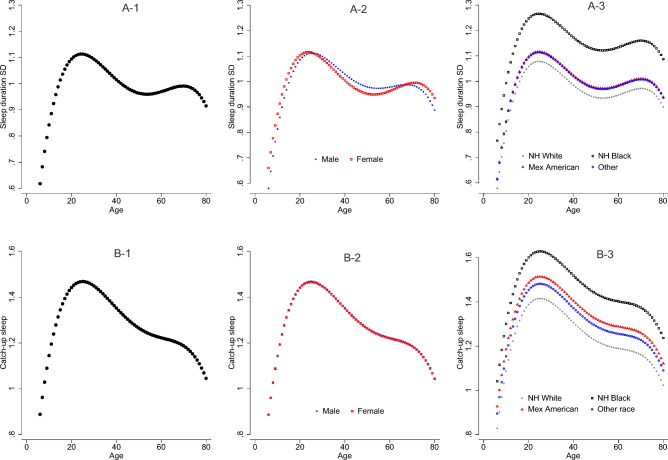
Age, sex, and race distribution of sleep duration SD and catch-up sleep. The unit for Y-axis is hours. (**A**) 1–3 for sleep duration SD. Please note the curve for Other race is overlapped with the curve of Mexican American. (**B**) 1–3 for catch-up sleep.

**Figure 3:**
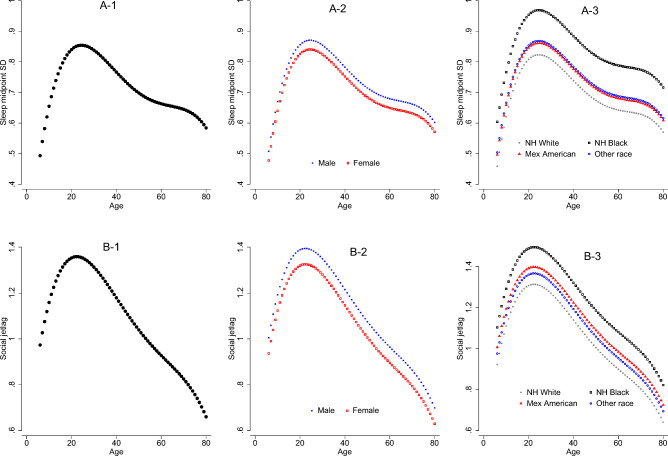
Age, sex and race distribution of sleep midpoint SD and social jetlag. The unit for Y-axis is hours. (**A**) 1–3 for sleep midpoint SD. Please note the curve for Other race is overlapped with the curve of Mexican American. (**B**) 1–3 for social jetlag.

Figure 2 displays the changes in the sleep duration SD and catch-up sleep with age and possible effect modifiers of sex and race. Sleep duration SD increased until its first peak around age 20 then gradually declined until around age 50. After age 50, it showed a small increase again until around 70 to where it declined once more (Fig. 2A-1). There was no overall main effect of sex on sleep duration SD but there was a significant interaction between sex and age2. As shown in Fig. 2A-2, the decline in sleep duration SD from around age 20 to age 40 was larger in females. Furthermore, there was a significant main effect of race (*p* < 0.001) as NH Blacks had the highest median sleep duration SD of 64 min compared to 53 min *(p* < 0.001), 55 min (*p* < 0.001), and 55 min (*p* < 0.001) for NH Whites, Mexican Americans, and other races respectively (Fig. 2A-3). There were no significant interactions between race and age observed for sleep duration SD.

Catch-up sleep followed a similar pattern as sleep duration SD as it increased till around age 20 then gradually declined throughout with a small plateau between ages 50 and 70 (Fig. 2B-1). There was no overall main effect of sex on catch-up sleep and there were no significant interactions between sex and age observed (Fig. 2B-2). However, there was a significant main effect of race (*p* < 0.001). NH Blacks had the highest median catch-up sleep of 73 min compared to 58 min *(p* < 0.001), 66 min (*p* = 0.062), and 65 min (*p* = 0.019) for NH Whites, Mexican Americans, and other races respectively (Fig. 2B-3). In addition, the difference between Mexican American and NH Whites in catch-up sleep also reached significance (*p* = 0.013). There were no significant interactions between race and age observed for catch-up sleep.

Figure 3 displays the changes in the sleep midpoint SD and social jetlag with age and possible effect modifiers of sex and race. As shown in Fig. 3A-1, sleep midpoint SD increased with age until its peak around age 20. After age 20, sleep midpoint SD gradually declined until around age 50 where it leveled off until around age 70 before declining once again. There was a significant main effect of sex (*p* = 0.011) with males averaging 2 more minutes of midpoint of sleep SD compared to females (Fig. 3A-2). There was also a significant main effect of race (*p* < 0.001) observed with NH Blacks averaging 9 more minutes of midpoint of sleep SD compared to NH Whites (*p* < 0.001) and 5 more minutes compared to Mexican Americans (*p* < 0.001) and other races (*p* < 0.001) (Fig. 3A-3). There were no significant interactions between race and age observed.

As shown in Fig. 3B-1, social jetlag is present in early ages beginning almost at an average of one hour around age 6 and gradually increasing until around age 20. After around age 20, social jet lag steadily declines with age. There was a main effect of sex (*p* = 0.006) with males averaging 5 more minutes than females (Fig. 3B-2). There were no interactions between sex and age found. However, there also was a main effect of race (*p* = 0.001) which was driven by the significant difference (*p* < 0.001) between NH Blacks and NH Whites with NH Blacks averaging 12 more minutes of social jet lag compared to NH Whites (Fig. 3B-3). Mexican Americans and other races had higher values than NH Whites and lower values than NH Blacks in social jetlag, and the differences were significant in the comparisons with NH Blacks (*p* = 0.039 for Mexican Americans and *p* = 0.018 for other races). There were no interactions observed between race and age.

In the sensitivity analyses of excluding participants with short duration of accelerometer recording, the results remained the same (Supplementary Table S5, Table S6, Figure S1 and Figure S2).

## Discussion

Within our study, we used actigraphy data to provide objective estimates of multiple sleep variability parameters: sleep duration SD, catch-up sleep, sleep midpoint SD, and social jetlag. Our study is the first to show the distribution of these parameters in a nationally representative sample of the United States. We explored the differences in these parameters of sleep variability across the life span as well as the potential modifying effects of sex and race. We found that American youth and young adults as well as NH Blacks had the largest variability across all sleep variability parameters compared to the other age groups or the other racial groups. The high rates of sleep variability in youth and young adults is especially concerning given its relationship with suicidal ideation and the observation that suicide death is the second leading cause of death among youth in the US^19^.

American young adults aged 18–25 had the highest sleep duration SD averaging 70 min which is consistent with a recent study on eight-pooled datasets which found that the 18–24 age group had the highest sleep duration SD averaging 77 min in the datasets using accelerometer recording^20^. Additionally, children aged 6–9 and 10–13 had lower rates of sleep duration SD when compared to post-pubertal adolescents (ages 14–17). Research from a 2011 review paper delved into the intricate connection between bioregulatory mechanisms and psychosocial factors on worsened sleep patterns in teenagers^21^. The findings of this work suggested that maturation leads to a delayed shift in adolescents’ circadian rhythms in addition to increased evening alertness^21,22^. Additionally, children growing older is associated with less strict parent-set bedtimes, increased screen time, and greater academic demands^22^. Unfortunately, this shift towards later bedtimes is often not matched with later wake times, as school start times remain relatively early. The interplay between all of these factors could underlie increasing sleep duration SD through adolescent maturation. Substance usage like caffeine, alcohol, and marijuana might also play a role, particularly in young adults. Although limited, studies on sleep schedule regularity and academic performance in adolescents and young adults indicate that a higher index of sleep regularity is associated with better academic performance^23,24^. Notably, students with failing grades reported greater differences between weekend and weekday sleep schedules^23^. This emphasizes the importance of further research on sleep variability in children and young adults.

Sleep duration SD showed a bimodal distribution with peaks around ages 20 and 70. This distribution held true when accounting for the impact of sex and race. Aging is associated with increased prevalence of multiple components which impact sleep such as multimorbidity, polypharmacy, psychosocial factors and many primary sleep disorders. All of which could contribute to increasing sleep duration SD after age 50^25^. The average age of retirement in 2013 was around 63 years old which also factors in as adults may taper off working hours leading into official retirement^26^.

The Multi-Ethnic Study of Atherosclerosis (MESA) in 1922 participants aged 45–84 found that almost 40% participants had > 90 min of sleep duration SD and these participants had higher averages of BMI, blood pressure, and diabetes prevalence compared to those with < 90 min of sleep duration SD^4^. After adjusting for cardiovascular disease risk factors, this study also found that every 1 h increase in sleep duration SD was associated with a 36% higher rate of cardiovascular risk^4^. Of the 4125 participants over the age of 45 within the current study only 14% had > 90 min of sleep duration SD. In addition to our study being conducted in a national representative sample, another potential reason for this difference could be that the previous study had different racial demographics. Of the participants of MESA 28% were African American compared to our study which had 11% NH Black participants. As shown in our study and prior research, NH Black Americans have greater amounts of sleep variability compared to other racial categories. Regardless, these findings illustrate the important impact of increasing sleep duration SD on the health of the geriatric population and emphases the need for ensuring public health education targeting on this topic. In addition, most research on the negative health impacts of sleep variability focus on older adults; however, our finding that 16% of participants aged 6–9 and 32% of participants aged 10–13 had 59 min of sleep duration SD highlights that variability in sleep pattern begins early in childhood. This warrants longitudinal studies to understand the impact of chronic sleep variability in childhood on different health parameters in later life.

In addition, our study examined the distribution of catch-up sleep across the lifespan. We found that 25% of participants aged 14–17 had greater than 2 h of catch-up sleep which aligned with a previous study that reported 25.5% of adolescents in their study had > 120 min of catch-up sleep^27^. Prior literature has demonstrated that amounts of weekend catch-up sleep greater than 2 h may be associated with mood and behavior disorders in adolescents^28^. Additional research is needed to understand the impact of different amounts of catch-up sleep on health through the lifespan.

Many factors like work and school schedules cause individuals to wake and sleep earlier during weekdays compared to weekends. This discrepancy is referred as social jetlag. We explored the distribution of social jetlag across all age groups. We found that social jetlag peaked around age 20 but all ages had varying amounts of social jetlag even young children. There is limited data on the quantity of social jetlag in various age groups; however, there have been studies which assessed total social jetlag in adolescents and in adults. A previous study used questionnaires to quantify social jetlag in non-shift workers aged 18–78 and found that almost 30% of participants had 1 to 2 h of social jetlag^29^. In our study, the average social jetlag for participants aged 18–78 was 64 min with 26% experiencing ≥ 1.5 h social jetlag. In addition, a study of undergraduate students which used actigraphy estimated that students averaged about 40 min of social jetlag^30^. This is lower than our finding of an average of 1 h 12 min of social jetlag in the age group of 18–25 in our study. This discrepancy could be related to the small sample size (n = 84) of the earlier study and all the participants being university students. A recent study found that greater social jetlag was associated with lower semester GPAs in older adolescents further emphasizing the link between sleep variability and academic performance in youth^31^. Moreover, our investigation into sleep variability trends within a large United States cohort exhibits alignment with analogous cross-sectional studies in other countries. A study of Australian children (*n* = 4032) between 9 and 18 years old delineated that older age groups had more pronounced differences in sleep–wake patterns on weekdays to weekends^32^. This result was supported by a study in Germany exhibiting lower levels of social jetlag in younger children aged 5 to 7 when compared to older children aged 8–20^33^. Further research should be conducted to explore how sleep variability trends in the United States relate to other countries as cultural differences likely influence sleep patterns, especially in children and youth^34,35^. These cultural differences could include meal times, family routines, or duration of daily schooling. For example, youth in Croatia attend school in the afternoon to accommodate work schedules^36^. Differing societal stressors including political or economic turmoil could also alter sleep patterns and should be considered.

Similar to the findings from the study on the eight-pooled datasets, we did not find a consistent difference between males and females in sleep variability parameters^20^. Sleep duration SD and catch-up sleep did not show significant differences between male and female; however, there was a significant main effect of sex on sleep midpoint SD and social jetlag as males averaged greater amounts of each compared to females.

Current literature is growing on sleep through a social-environmental perspective. Multiple studies have indicated that race, as a social category, impacts sleep quality and quantity across the lifespan implicating race as a critical factor in current sleep disparities^37–39^. Many of these studies focused on comparisons between NH Black Americans and NH White Americans. However, as a nationally representative US sample, our study consisted of 65% NH White Americans, 11% NH Black Americans, 10% Mexican Americans and 14% of others. This allowed for reporting of sleep variability characteristics in Mexican Americans which is rarely cited in current research. We observed that Mexican Americans had significantly lower amounts of sleep duration SD, sleep midpoint SD and social jetlag compared to NH Blacks. Mexican Americans also had significantly higher amounts of catch-up sleep compared to NH Whites. In line with previous reports, NH Black Americans had worse sleep variability compared to all other racial groups. These findings align with a previous study which found that NH White youth had more sufficient sleep compared to NH Black youth^38^. A multitude of factors may underlie the reasoning for these results. For instance, shift work tends to have variable shifts which could impact sleep variability, and people from minority racial group disproportionately hold shift work positions^39^. In addition, minority racial groups undergo an increased amount of daily stressors comparatively which is a potential underlying factor as stress is known to disrupt sleep patterns^39^. Moreover, a recent actigraphy study explored the relationship between racial disparities in sleep characteristics and hypertension prevalence and concluded that sleep maintenance mediated over 11% of the difference in hypertension prevalence between NH Blacks and NH Whites within their sample^40^. These findings highlight the significance for multifaceted approaches that include racial disparity when studying the impacts of sleep on health outcomes.

There are some limitations to this study. First, although accelerometer recordings provide objective measurements of sleep parameters, this method cannot detect a difference between lying still while awake or being asleep. Second, occupation status was not obtained from participants in the NHANES 2011–2014 cycle, therefore, exclusion criteria for certain occupations like shift workers could not be performed. Third, the accelerometer data were obtained during the period of 2011–2014 and these accelerometer-data based sleep parameters might not represent the sleep measurements of the current US population. More recent accelerometer data from national surveys are warranted. Forth, calculating parameters of sleep variability usually requires a weeklong accelerometer recording^41^. Although 7-day accelerometer data were available for the NHANES 2011–2014 cycle data, after preprocessing and quality control, we kept the participants with at least 4 days (with at least one day recorded during the weekend) of high-quality accelerometer data to maximize the sample size. In the sensitive analyses, we repeated the analyses in these participants with at least 6 days high-quality accelerometer data and observed the same results, indicating that the small percentage of the participants with a relatively short period of qualified accelerometer recording does not affect the findings of this study. On the other hand, the study by Fischer et al.^41^ also showed that sleep regularity index (SRI) displayed the least dependence on the length of the availability of accelerometer recordings. SRI measures the similarity of an individual’s sleep–wake patterns from one day to the next, based on binary sleep–wake state classifications. It calculates the percentage probability of an individual being in the same state (sleep vs. wake) at any two time points 24-h apart, averaged across the study. The SRI is scaled to range from 0 (random) to 100 (perfectly regular). We repeated the analysis using SRI as an index of sleep regularity (Supplementary Figure S3) and observed the similar findings as the other sleep variability parameters with males and NH Black participants having lower SRI than females and NH White participants and the lowest SRI displaying at age 18–25. This further supported that our findings were not biased by the inclusion of participants with relatively short duration of accelerometer recordings. However, future studies with longer recording periods are still recommended, especially for catch-up sleep and social jetlag for which 14 days of accelerometer recording capturing 2 weekends will be able to provide more reliable findings. Fifth, the minute summary count of accelerometer data was used for the detection of the sleep period time window (i.e. the block of the longest sleep period in the day [noon–noon]), therefore the napping period was not captured. Further studies are warranted to explore how napping impacts sleep variability parameters. Another limitation arises from the inability to account for environmental factors that may alter sleep habits, particularly when minority populations are often more susceptible to these external determinants potentially worsening their sleep outcomes. Finally, as the findings are based on a cross-sectional analysis, temporal comparisons are limited.

In conclusion, for the first time our study provides the distribution of multiple objectively measured sleep variability parameters over lifespan by sex and race in a US nationally representative sample. This study not only provided solid evidence on the high prevalence of irregular sleep patterns in the US population, but also indicated that irregular sleep begins in childhood and shows racial disparity. These findings provide understanding of sleep patterns for residents of the United States as well as emphasize the importance in future research on the social determinants of sleep from multiple aspects. Sleep variability is likely a modifiable risk factor for a variety of health conditions. This information can also further research and public health agendas on sleep hygiene.

### Supplementary Information

Supplementary Information.

## Data Availability

All data are publicly and freely available from the US Centers for Disease Control and Prevention’s National Center for Health Statistics and can be accessed at https://www.cdc.gov/nchs/nhanes/index.htm.
