# Supplementary material for: Age, sex and race distribution of accelerometer-derived sleep variability in US school-aged children and adults
Source: Sci Rep. 2023 Dec 13;13:22114. doi: 10.1038/s41598-023-49484-5 (PMC10719297; doi:10.1038/s41598-023-49484-5)
Supplement: Supplementary file 1 — Supplementary Information. [file 41598_2023_49484_MOESM1_ESM.docx]

**Supplementary document**

**Age, sex and race distribution of accelerometer-derived sleep variability in US school-aged children and adults**

Elexis Price^a^, Xinyue Li^b^, Yanyan Xu^c^, Asifhusen Mansuri^d^, William V. McCall^e^, Shaoyong Su, ^c^, Xiaoling Wang^c *^

^a^ Medical College of Georgia, Augusta University, Augusta, GA, USA

^b^ School of Data Science, City University of Hong Kong, Hong Kong, China.

^c^ Georgia Prevention Institute, Medical College of Georgia, Augusta University, Augusta, GA, USA

^d^ Division of Pediatric Nephrology and Hypertension, Children’s Hospital of Georgia, Medical College of Georgia, Augusta University, Augusta, GA, USA

^e^ Department of Psychiatry and Health Behavior, Medical College of Georgia, Augusta University, Augusta, GA, USA

Address for correspondence and reprints:

Xiaoling Wang

Georgia Prevention Institute,

Medical College of Georgia,

Building HS-1721

Augusta, GA 30912, USA

**Accelerometer recording and data preprocessing**

All participants aged 6 years and older during the 2011-2012 cycle and all participants aged 3 years and older during the 2013-2014 cycle were asked to wear an accelerometer (ActiGraph Model GT3X+, ActiGraph of Pensacola, FL) all day and night for 7 consecutive days. The device was worn on the non-dominant wrist, if possible. Raw signals obtained on the x-, y-, or z-axes every 1/80 of a second (80 Hz) were processed, flagged and summarized at the minute level and released by NHANES in November 2020. These summary measures in the minute summary file (PAXMIN) are specified in Monitor-Independent Movement Summary (MIMS) units, which is a non-proprietary, open-source, device-independent universal summary metric developed by researchers at Northeastern University ^1^. MIMS triaxial value (variable name: PAXMTSM) at the every minute level was used to calculate sleep parameters. MIMS triaxial values were changed to missing (i.e. a value of 0) if they met any of the following conditions: (1) PAXMTSM is coded as "-0.01"; (2) estimated wake/sleep/wear status during the minute (variable name PAXPREDM) is coded as “Non wear”; or (3) minute data quality flag count (variable name PAXQFM) is larger than “0”. R package “accelmissing” ^2^ was used to impute the missing count values in the accelerometer data with the following pre-processing steps: (1) the minimum minutes of missing interval were defined as 60 minutes; (2) the valid days were defined as more than 16 hours of wearing; and (3) the minimum number of valid days that the subject should have was defined as 4 days.

1. John D, Tang Q, Albinali F, Intille S. An Open-Source Monitor-Independent Movement Summary for Accelerometer Data Processing. J Meas Phys Behav. 2019 Dec;2(4):268-281. doi: 10.1123/jmpb.2018-0068. PMID: 34308270; PMCID: PMC8301210.
2. Lee JA, Gill J. Missing value imputation for physical activity data measured by accelerometer. Stat Methods Med Res. 2018 Feb;27(2):490-506. doi: 10.1177/0962280216633248. Epub 2016 Mar 17. PMID: 26994215.

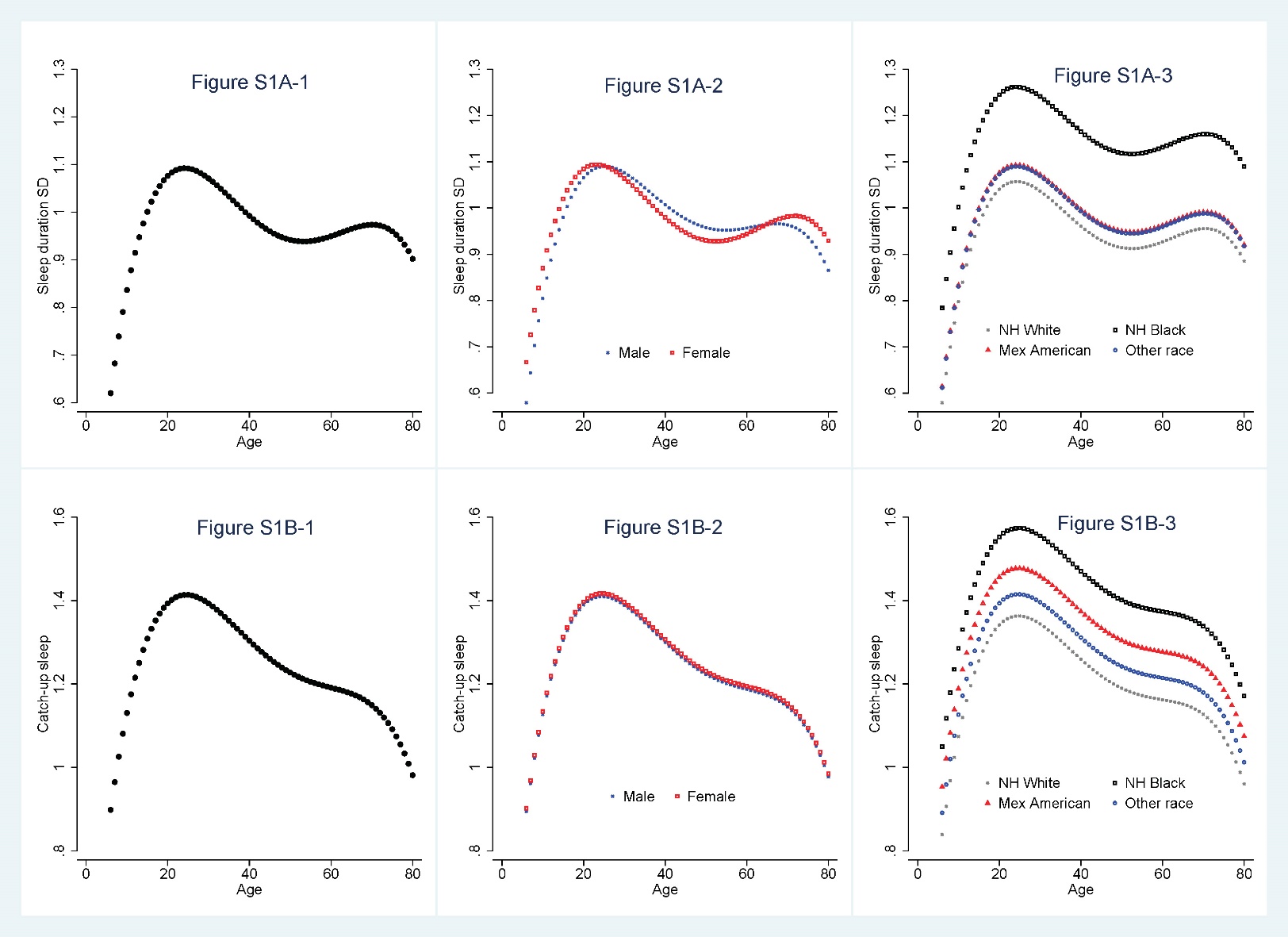


**Figure S1**. Age, sex, and race distribution of sleep duration SD and catch-up sleep in participants with ≥6 day accelerometer data. The unit for Y-axis is hours. (A) 1-3 for sleep duration SD. Please note the curve for Other race is overlapped with the curve of Mexican American. (B) 1-3 for catch-up sleep.


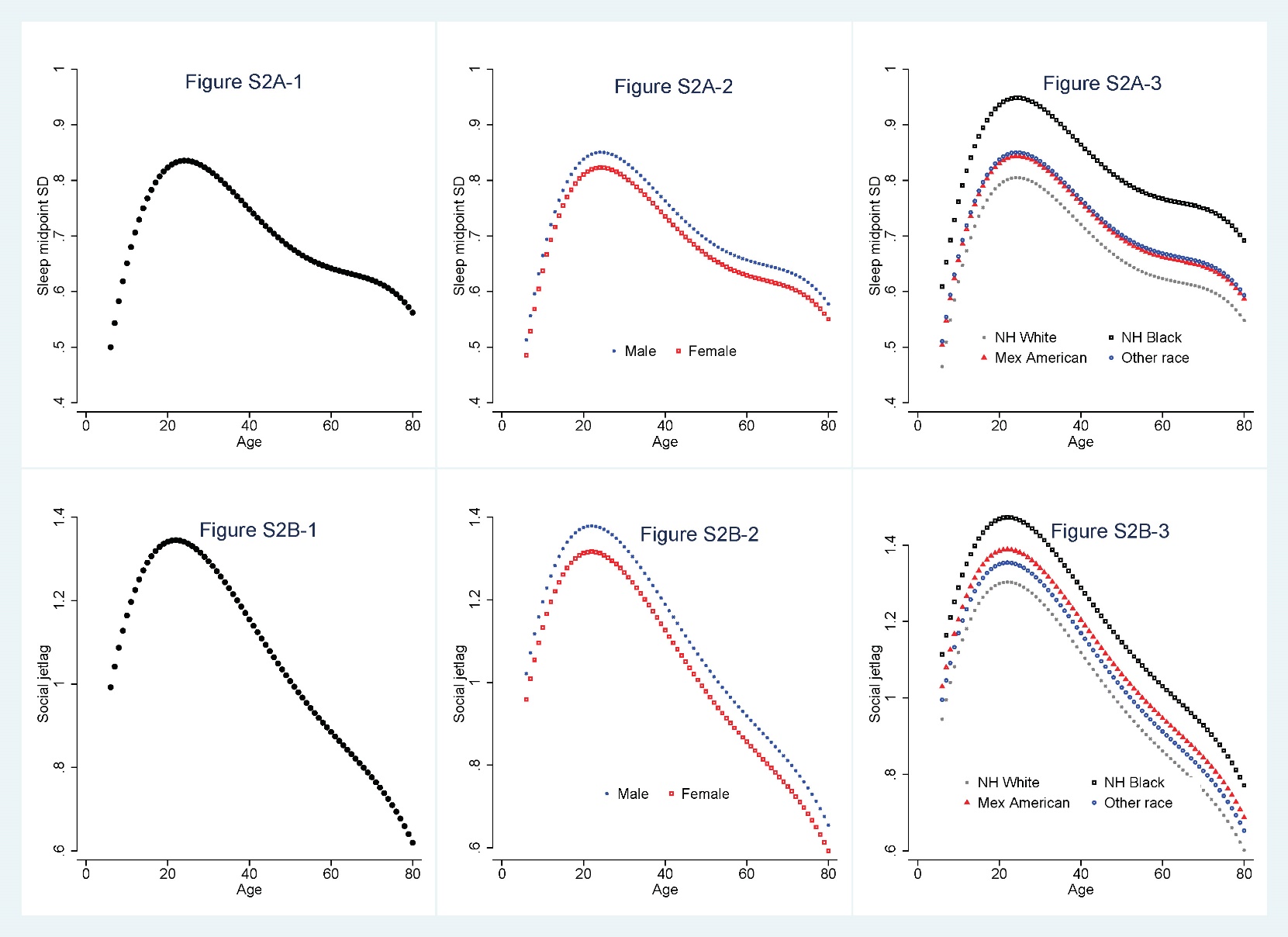


**Figure S2**. Age, sex and race distribution of sleep midpoint SD and social jetlag in participants with ≥6 day accelerometer data. The unit for Y-axis is hours. (A) 1-3 for sleep midpoint SD. Please note the curve for Other race is overlapped with the curve of Mexican American. (B) 1-3 for social jetlag.


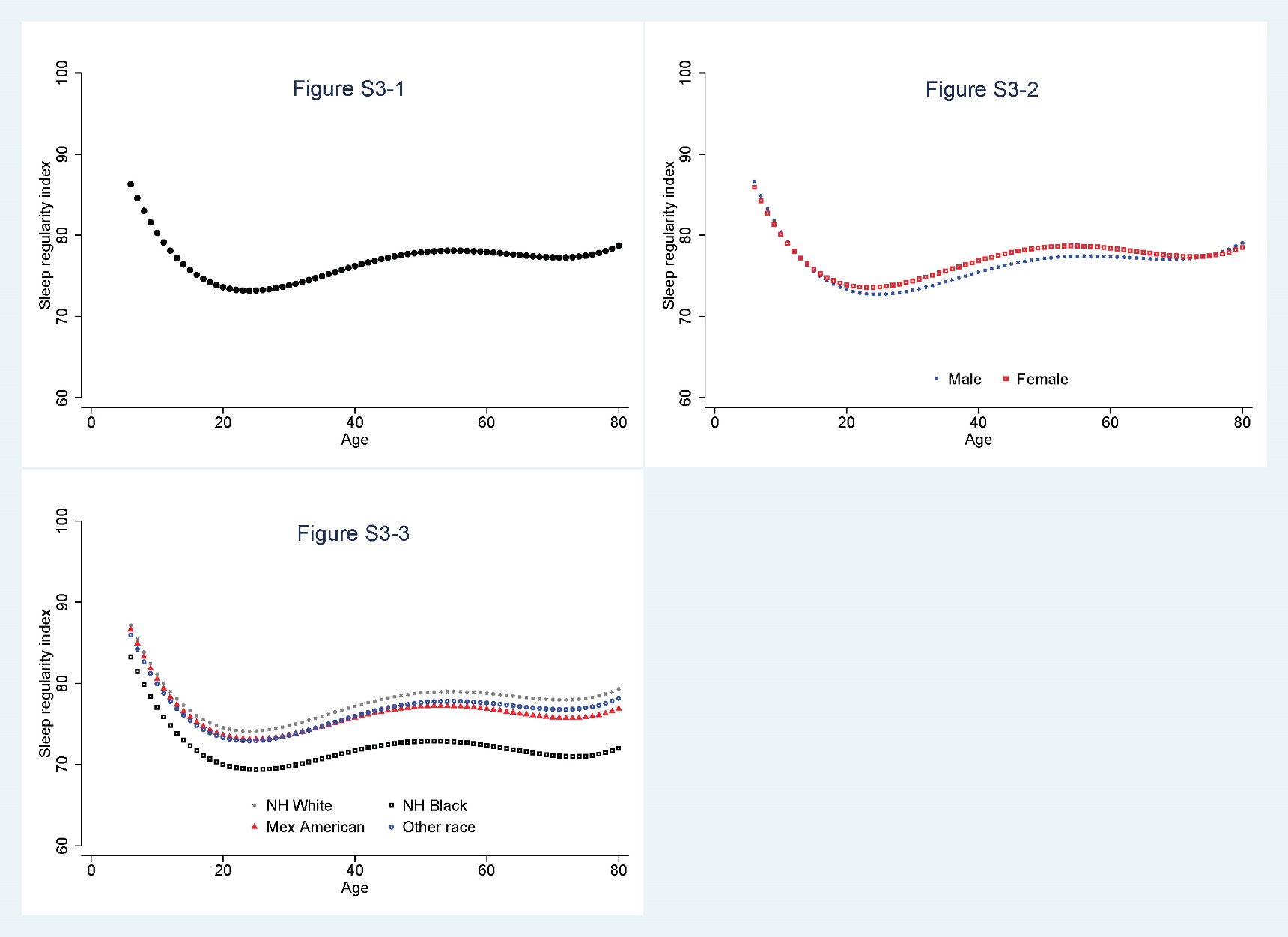


**Figure S3**. Age, sex and race distribution of Sleep regularity index.
